# Dual Annual Spawning Races in Atlantic Sturgeon

**DOI:** 10.1371/journal.pone.0128234

**Published:** 2015-05-28

**Authors:** Matthew T. Balazik, John A. Musick

**Affiliations:** 1 Center for Environmental Studies, Virginia Commonwealth University, 1000 West Cary Street, Richmond, Virginia, United States of America; 2 Virginia Institute of Marine Science, College of William & Mary, P.O. Box 1346, Gloucester Point, Virginia, United States of America; Aristotle University of Thessaloniki, GREECE

## Abstract

Atlantic sturgeon (*Acipenser oxyrinchus oxyrinchus*, Acipenseridae) populations in the United States were listed as either endangered or threatened under the Endangered Species Act in 2012. Because of the endangered/threatened status, a better understanding of Atlantic sturgeon life-history behavior and habitat use is important for effective management. It has been widely documented that Atlantic sturgeon reproduction occurs from late winter to early summer, varying clinally with latitude. However, recent data show Atlantic sturgeon also spawn later in the year. The group that spawns later in the year seems to be completely separate from the spring spawning run. Recognition of the later spawning season has drastically modified estimates of the population status of Atlantic sturgeon in Virginia. With the combination of new telemetry data and historical documentation we describe a dual spawning strategy that likely occurs in various degrees along most, if not all, of the Atlantic sturgeon's range. Using new data combined with historical sources, a new spawning strategy emerges which managers and researchers should note when determining the status of Atlantic sturgeon populations and implementing conservation measures.

## Introduction

Sturgeon species (family Acipenseridae) are generally threatened along their entire range around the northern hemisphere [1, 2]. The Atlantic sturgeon (*Acipenser oxyrinchus oxyrinchus*, Acipenseridae) occupies the Atlantic slope of North America and five distinct population segments were listed as either endangered or threatened under the U.S. Endangered Species Act in 2012 [3, 4]. Because of the endangered/threatened status, a better understanding of life-history traits and habitat use are necessary for more effective management. Over the past decades several reviews about the status and life history of Atlantic sturgeon (AS) have been published [5, 6, 7, 8]. The general consensus has been that AS spawning varies clinally along its range with spawning occurring in the spring around March in the Savannah River, GA, while progressing temporally to July in the St. Lawrence River in Quebec. Temporal spawning estimates have been derived mostly from historical fisheries data [9, 10, 11]. Historical fisheries targeted flesh and roe, the latter being more valuable [11, 12]. Since the collapse of AS populations in the late 19^th^ century, due mostly to over-exploitation [13], very little research on the life history of the species was conducted from 1900 to 1980.

Sturgeon species show high plasticity in regards to spawning behavior. Berg [14] did an extensive overview of multiple seasonal spawning races in anadromous fishes around the world. Multiple spawning races (or runs) occur when a single species has distinctly separate groups spawning in the same river system which we term “dual spawning”. Berg [14, 15] describes groups as vernal and hiemal spawning races. Distinctly separate spawning groups have been described for several Acipenseriformes around the world. Dual spawning occurs in many Eurasian sturgeon species. The Black and Caspian Seas have several species with vernal and hiemal races: Beluga Sturgeon (*Huso huso*), Russian Sturgeon (A. *gueldenstaedtii*), Ship Sturgeon (*A*. *nudiventris*), Stellate Sturgeon (*A*. *stellatus*), and Sterlet Sturgeon (*A*. *ruthenus*). The vernal group enters the river during the spring with almost ripe gonads and spawns soon after entering the river. The hiemal group enters the river the same time or just after the vernal group but with non-ripe oocytes. The hiemal group with non-ripe oocytes overwinter in the river while the oocytes develop and the fish spawns the subsequent spring. The males follow a similar pattern [14, 15, 16]. The hiemal groups historically spawned further upstream than the spring groups, but dams have restricted access to most upstream spawning habitats [15, 16]. The Kaluga Sturgeon (*H*. *dauricus*) in the Amur River, Russia, has a spawning pattern similar to the pattern just previously described [15]. However, in addition to the vernal and hiemal migratory groups, the Kaluga Sturgeon in the Amur River also has a resident population that never descends to the sea. Individuals of the resident Kaluga Sturgeon population are larger than the anadromous races [15]. The Siberian Sturgeon (*A*. *baerii*) also has a resident and anadromous race in Siberian River systems [16]. In the southern portion of the Caspian Sea, two groups of Persian Sturgeon (*A*. *persicus*) enter the rivers in April and May. One group enters the river with mature gonads and spawns from April to June like the vernal groups previously described. The hiemal group arrives with less developed gonads, but instead of overwintering, the group spawns in August and September. Some post-spawn Persian Sturgeon will overwinter in the rivers before migrating back to the Caspian Sea. Persian Sturgeon in the northern region of the Caspian Sea follow the typical vernal/hiemal spawning pattern with an overwintering stage [14, 15, 16]. The various reproduction patterns of Persian Sturgeon show the same species may utilize disparate spawning strategies in different regions. Based on captures of ripe fish, Yang [17] and Zen [18] suggested that Dabry’s Sturgeon (*A*. *dabryanus*) in the Yangtze River Basin, China, spawn both in the spring and the fall [19]. Data are scarce in regards to Adriatic Sturgeon (*A*. *narracii*) and Common Sturgeon (*A*. *sturio*) reproduction; however, both species show potential signs of dual spawning behavior [14, 15, 16, 20].

Dual spawning races likely occur in some North American sturgeon species. In the Suwannee River, Florida, Sulak and Clugston [21] verified Gulf Sturgeon (*A*. *o*. *desotoi*, a subspecies of AS) spawn in March and April, by collecting ova on eggs mats. Sulak and Clugston [21] also captured fully ripe females and males with motile sperm along with juveniles of certain sizes that suggest Gulf Sturgeon also spawn during October and November. Continued research in the Suwannee River by Randall and Sulak [22] described suspected spawning behavior of the October and November group using telemetry movements of adult male fish. Lake Sturgeon (*A*. *fulvescens*) appear to have two distinct groups spawning during the spring without an overwintering period. In the Black River, MI, the same individuals of one group repeatedly spawn about 3 weeks earlier and further upstream than another group [23].

In regards to AS spawning we use the terms spring (vernal) to designate the earlier spawning race/group and fall (hiemal) for the later spawning race/group even though the actual time of spawning may not occur during the spring or fall season. It has been widely documented that Atlantic sturgeon reproduction occurs from late winter to early summer, varying clinally with latitude [5, 6, 8]. However, recent data show Atlantic sturgeon also spawn during the late summer/early fall, completely separate from the spring spawning run. Fall spawning has been confirmed in the Roanoke River, NC by collection of fertilized eggs [24], and strong empirical evidence of AS fall spawning has been noted in the James River, Virginia [25], South Carolina [26, 27], and Georgia (Douglas Peterson, University of Georgia, personal communication). There is debate whether "fall" spawning is a new phenomenon or something that has been overlooked due to life history, harvest techniques and/or low population numbers. Here we present new data to further support the fall spawning data in Balazik et al. [25] along with data suggesting spring spawning still occurs in the James River. We also subject historical reports about AS spawning to greater scrutiny to show that fall spawning has been occurring since European arrival to the North America, that the fall group is possibly the principal spawning race historically and presently sustaining the James River population, and that dual spawning may be occurring along the fish’s entire range.

## Study Area

**Fig 1:**
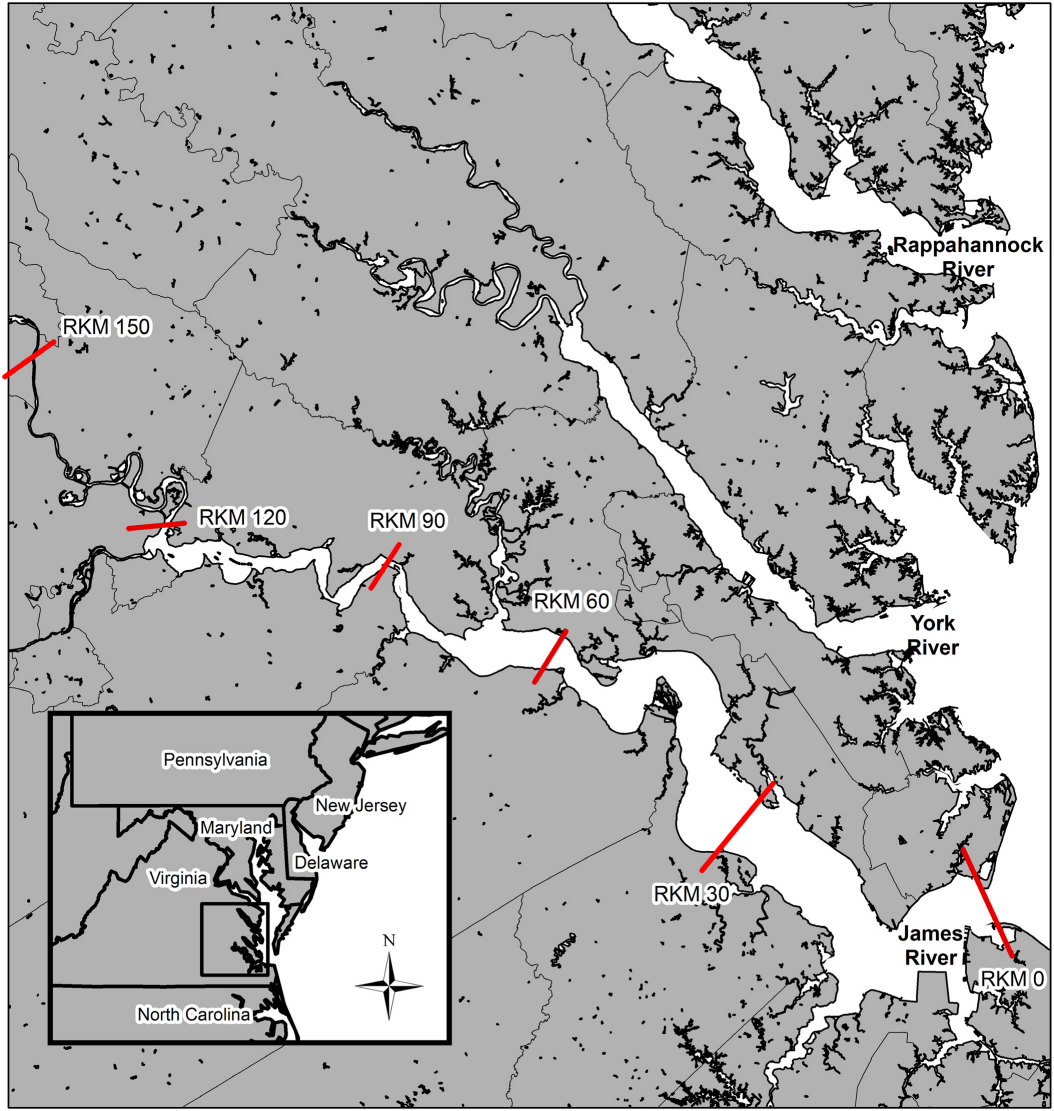
Map showing the tidal portion of the James River with river kilometer (rkm) designations.

The James River, river mouth located at 36.98891, -76.30362, is the southern-most major tributary of the Chesapeake Bay (Fig 1). The James River is 696 km long and drains 26,164 km^2^. The tidal portion extends up to Richmond, VA located at rkm 155. River width varies between 0.7 and 7.1km up to rkm 120 and then narrows (range: 0.1 to 0.4 km). The federal navigation channel maintained by the U.S. Army Corps of Engineers runs from the river mouth at Hampton Roads, VA to rkm 150 and is maintained to a minimum depth of 7.6 m and a minimum width of 91.4 m.

## Methods

This study was carried out following guidelines set by Virginia Commonwealth University’s Institutional Animal Care and Use Committee (#AD20127) and National Marine Fisheries Service endangered species permit (#16547). All efforts were made to minimize stress to the fish. Adult AS were collected by gill net from the James River to examine for evidence of spawning from March to May 2008–2014 and August to October 2008–2013. Results of fall sampling for 2008–2011 were reported by Balazik et al. [25] along with details of the methodology which was also used in the present study. From 2008 to 2010 all sampling occurred around rkm 120 [25]. In response to observations of AS breaching at rkm 90 and discovery of a post-spawn female at rkm 91, spring efforts were moved downstream in May 2011.

During 2012 and 2013 spring sampling occurred between rkm 87 and 97. In the spring of 2014 sampling also occurred at rkm 33. Spring sampling was between 1 April and 20 May. Similar to previous years [25], fall sampling was conducted upstream of rkm 108 between 15 August and 20 October of 2012 and 2013 (Fig 1). Prior to 2014 gill nets varying from 25 cm to 33 cm stretch mesh, 3–5 m deep, were used during both seasons. In 2014 gill nets ranging from 25–46 cm stretch mesh and heights of 3–7 m deep were fished in the spring. All nets were 92 m long and anchored along the bottom. Set depths varied from 3–25 m during the spring and 3–12 m during the fall. Nets were fished following National Marine Fisheries Service Endangered Species Protocols (Permit # 16547). All fish were measured for fork length (fl) ± 1 cm and greatest girth ± 1 cm, and tagged with a T-Tag and a passive-integrative-transponder tag. If there were no previous tags found, new tags were placed. When possible, weight in kg (± 1 kg) was measured. Sex was usually determined visually by the observation of expressed reproductive material [25]. If reproductive material was not expressed fish were anesthetized using electronarcosis [25, 28] and gonads were viewed internally through an incision made for telemetry tag placement. Some fish were tagged externally or internally with Vemco ultrasonic telemetry tags. A more in-depth description of surgical techniques and fish behavior after tagging can be found in Balazik [28] and Balazik et al. [29]. One female was caught and tagged on 20 April 2011 at rkm 35 as part of another study.

From 2012 to 2014 telemetry movements in the James River were monitored using an array of about 40 Vemco VR2W receivers that covered from rkm 22 to rkm 142 in 2012, and from rkm 22 to rkm 155 in 2013 and 2014. Receivers were removed briefly for maintenance during the winter after all tagged adult fish had left the system.

## Results

During the study 369 (349 males, 4 females, and 16 unconfirmed) different adult AS were caught during the fall period and four were caught during the spring period. Of the unconfirmed fall fish four were likely females; the remaining 12 unconfirmed were likely males, but due to time restraints sex could not be verified [25]. Four adult males were caught during the spring and expelled milt during capture (Table 1). One male, 184 cm fork length (fl), was captured at rkm 94 on 8 May 2011 and did not have a telemetry tag placed. A 180 cm fl male was caught on 20 April 2012 at rkm 93, and a telemetry tag was attached externally. No adults were captured in the spring of 2013; however, numerous adult-size fish were seen breaching around rkm 90. Two adult males were captured in spring 2014. One was caught on 19 April (fl = 190 cm) at rkm 33 and typically would not be considered a spring spawn fish until after the fish returned in subsequent years to confirm spawning season. Because the fish caught on 19 April 2014 moved upstream and left the river similarly to other spring spawning males we are confident this was a spring-run male. A fish captured on 12 May 2014 (fl = 167) at rkm 94 had healing scrapes and abrasions that correspond with spawning [11, 25]. Both 2014 collections had telemetry tags placed internally. In addition, one male and two female mortalities were documented in the spring. A very recent post-spawn female (fl = 201) was found at rkm 93 on 11 May 2011 and another was found at the mouth of the James River (fl = 212) on 5 June 2013. One mature male (fl = 201) was found at rkm 97 on 17 April 2011. Spring males averaged 180 cm fl while fall males averaged 152 cm fl (130–183 cm) and 29 kg (19–52 kg) (Table 1). A female with a concave belly (slunker [11] was captured on 14 September 2012 at rkm 120. Due to her large size and noticing she was releasing a few eggs she was cut out of the net beside the boat. Another female (fl = 198) with ova separated from the ovaries was caught at rkm 120 on 6 September 2013. Ten ova from the 6 September 2013 female had an average diameter of 2.6 mm with a range of 2.4–2.8 mm. From 2011–2014 about 85% of captured males expelled sperm during capture, and the remaining 15% had sperm extracted via a catheter. During the study period nine males were recaptured and expelled sperm both times. All sperm samples tested had motility verified [25]. During the entire study period 155 fall males were tagged with Vemco ultrasonic telemetry tags.

**Table 1:** Comparison of spring and fall Atlantic Sturgeon collections in the James River from 2012 through the spring of 2014.

	2012		2013		2014
Season	Spring	Fall	Spring	Fall	Spring
Fish Caught	1	73	0	163	2
Telemetry Tags Placed	1	72	0	54	2
Recaptures	0	6	0	10	0
Collection Dates	Aug 20	Aug 18/Oct 13	0	Aug 16/Oct 4	Apr 19, May 12
River kilometer caught	93	118–133	0	118–121	33–95
Male Average Fork Length cm (min-max)	180	154 (131–177)	NA	150 (119–176)	179 (167–190)
Tag returns	0	13	1	59	1
Residence of Returns	NA/May 12	May 5/Nov 2	Apr 10/May 20	May 10/Nov 5	Apr 4/May 17
River kilometer reached	102	142+	127	155	120

Number of Atlantic Sturgeon caught, telemetry tags placed, recaptures from previous years, collection dates and locations, fork length in cm, telemetry tag returns with residence and river kilometer reached by returning telemetered fish.

**Fig 2:**
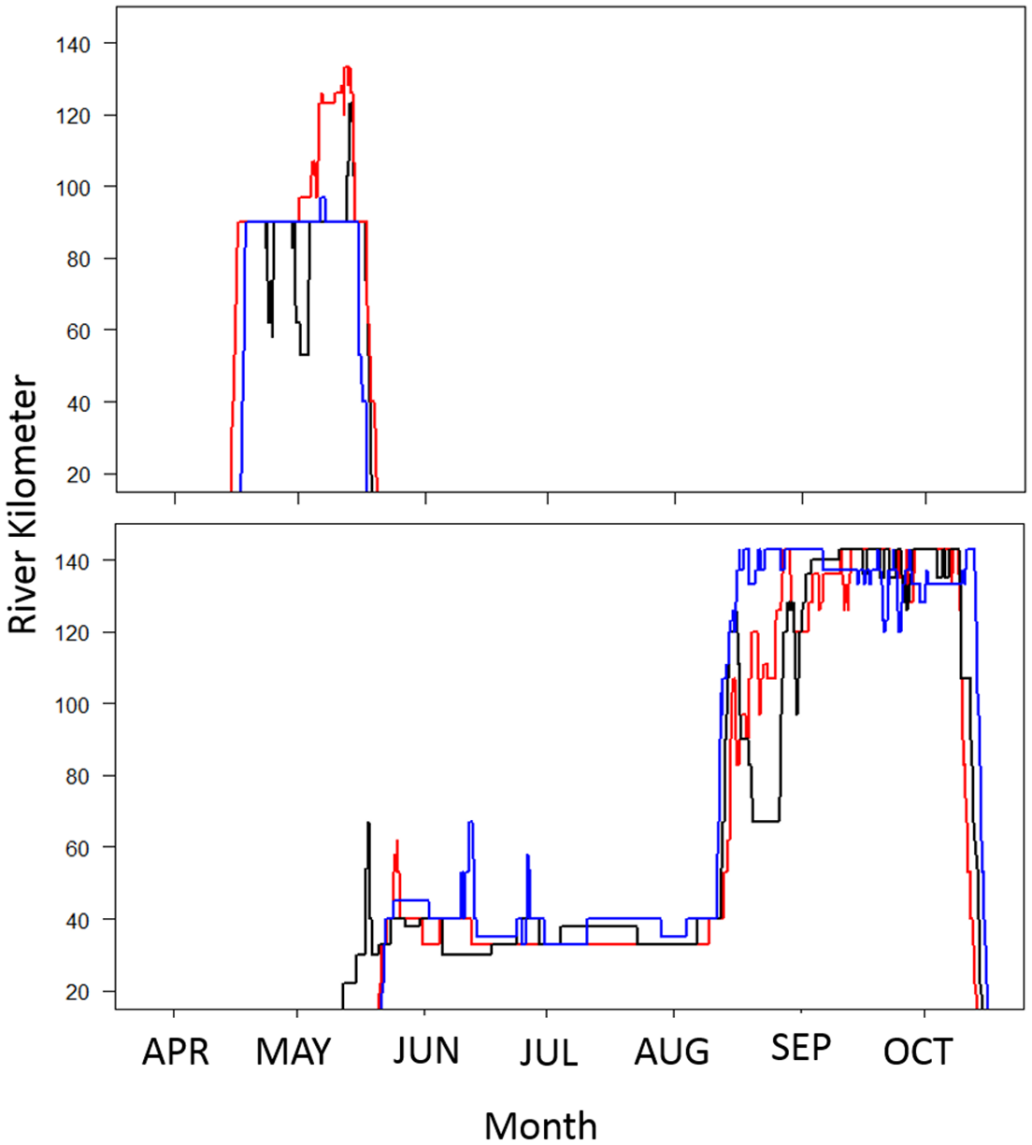
The telemetry movements (2012 black, 2013 red, and 2014 blue) of mature male Atlantic Sturgeon repeatedly returning to the James River, VA. The top panel (A) is the only telemetered spring sturgeon (tagged in 2012) and the bottom panel (B) is a representative displaying the typical behavior of fall-run sturgeon. The fall-run male was tagged in September of 2011 at rkm 120.

The one male telemetered in the spring of 2012 returned every year post-tagging (Fig 2). This spring male returned in early April, moved straight to hypothesized spawning grounds, and left the river by the end of May. The two males tagged in the spring of 2014 returned to hypothesized spawning ground in early April 2015. Telemetry data show that all of the 155 males tagged during the fall moved out of the river by November. Over half of the males caught upstream during the fall returned to the river during May and June (Fig 2). None of these moved up into the spawning reaches during the spring, but generally staged downstream (around rkm 40) in brackish water during the summer before moving upstream in August or early September (Fig 2). Some fish exhibited behavior very different from the general trend. For example a male tagged in 2011 returned each year during June, after the spring fish had left, and staged around rkm 107 from June to late August before moving upstream when the fish staging downstream moved upstream to spawning areas. Telemetered fish tagged during the fall continued to arrive at the staging area from July through August.

**Fig 3:**
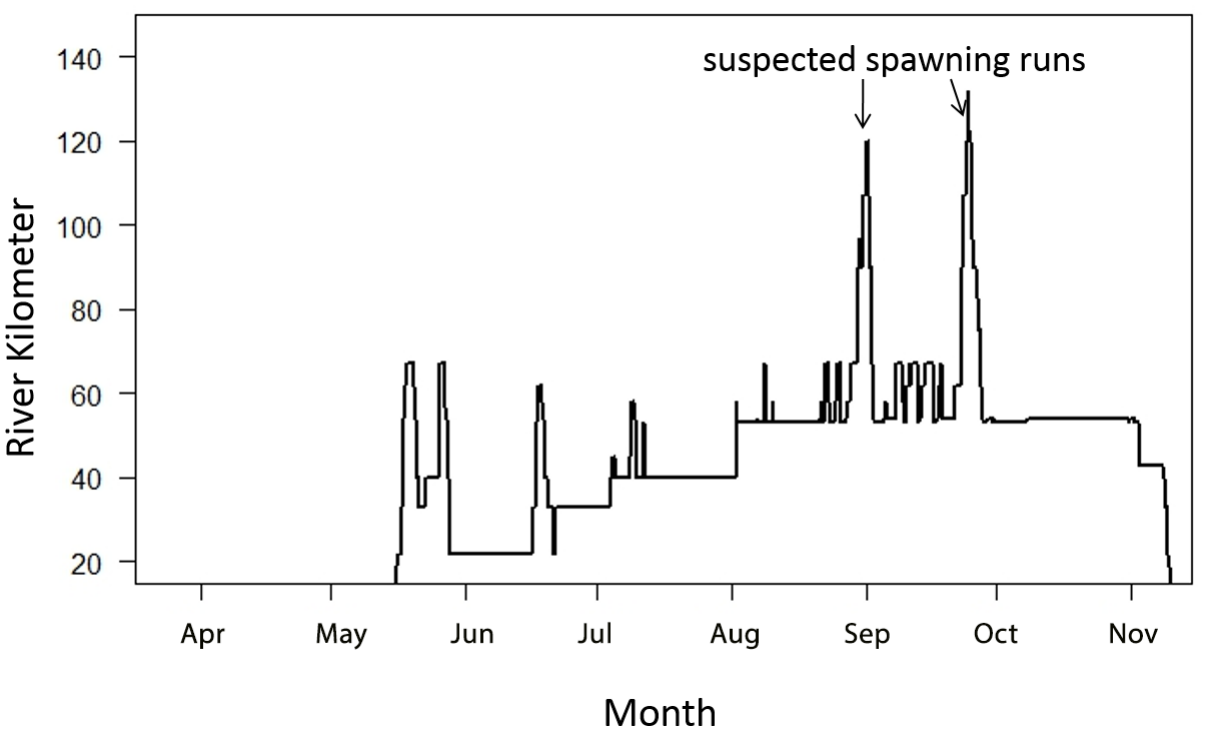
The 2013 telemetry movements of a female caught on 20 April 2011 at rkm 35. When captured her ova were immature and averaged 1.7 mm in diameter. The fish staged downstream in brackish water except for two suspected spawning runs in the in September. This female would have been susceptible to harvest as early as May when the historical fisheries were targeting what was thought as spring-spawn fish. Fall-spawning fish were likely harvested throughout the late spring and early summer which may be why the fall season was never officially documented in VA.

The female AS caught during another project on 20 April 2011 at rkm 35 returned to the James River 6 May 2013. The female staged between rkm 22 and 65 from May to August, spending considerable time around rkm 40 before making spawning runs in September (Fig 3).

## Discussion

Sturgeon exhibit considerable plasticity in regards to spawning behavior. Both species of the genus *Huso* and over half of *Acipenser* species utilize a dual-spawning strategy in some part of their range. Data suggest that AS reproduction behavior is similar to the Persian Sturgeon in the southern region of the Caspian Sea, the Darby’s Sturgeon in the Yangtze River basin, and other sturgeon species that have considerable gaps in information [14, 15, 16, 20].

While spawning has not been confirmed in the James River, strong empirical data suggests there are two separate spawning races. We think it is highly unlikely these adult anadromous fish return to freshwater each year expelling reproductive material without the intention of spawning. Telemetry data show two potential spawning runs: a spring run from late March to early May, and a fall run around September after an extended staging period in the lower river (Figs 2 and 3).

James River, VA: Because commercial harvest in the late 1800s decimated AS populations and due to lack of population recovery [8], AS life-history information has been scarce. Past reports stated AS spawning in the Chesapeake Bay occurs in April and May [5, 6, 7, 30]. These reports were made using data after the collapse of the AS population and none of the reports mention AS reproduction during August/September. Recent telemetry data show that two separate spawning runs likely occur in the James River (Fig 2): the spring group previously described in April and May, and the fall group from August to early October (Figs 2 and 3). The discovery of recently-spawned females and capture of mature males expelling milt suggests that AS reproduction still occurs in the James River during the spring (Fig 2). Unlike the spring group, the fall spawn group has a staging period in the river (Fig 2). Randall and Sulak [22] documented fall males having a long staging period in the Suwannee River, FL. While not officially described in the James River until relatively recently [25], colonial records suggest the fall group may have historically been the dominant race in the James River.

In 1607 Captain John Smith said “In summer no place affords more plenty of sturgeon”. John Smith also described catching sturgeon less than 0.9 m from late May to late June and sturgeon 1.8 to 2.7 m from July through September off Jamestown Colony located around rkm 50 [31, 32]. We assume these measurements were total length. The 1.8 to 2.7 m fish were likely fall-spawn fish. In 1612 William Strachey says “there is a ‘great store’ of sturgeon in May if spring comes early” [31]. In the 1630s David De Vries stated that a boat from England came to target sturgeon in the James River but failed because the best fishing was during the summer and the colonists could not preserve the sturgeon [31]. Most Virginia-colonial accounts describe great amounts of sturgeon during the summer months. The only Virginia-colonial account of sturgeon in March and April that we are aware of is by William Strachey in 1612 telling of catching sturgeon from the middle of March through August at the mouth of the James River [31]. It seems that the high abundance of AS was during the summer months when the fall group was staging in the river.

The fall spawning group in the James River may have never been described due to the timing of historical commercial fisheries. Coleman [33] described the AS fishery in the James River in the 1890s, writing that the commercial fishery operated along the north shore of the river. In June the fishery took place at Jamestown Island (~ rkm 50) and moved, likely upstream, as the season progressed. The peak of the fishery was in May/early June in the 1890s [33]. Seeley [34] noted catches of 300 fish a day occurred in some Virginian rivers during May, June, and July. There is no mention of large amounts of AS in April when the spring race entered the river.

Currently the spring-spawn AS leave the river by mid-May, and assuming timing has not been modified due to climate change the spring fish would only have been present during the early portion of the “peak” of historical fisheries. With commercial fishing occurring in the oligahaline portion of the river from May to July a significant portion of landings may have been fall spawning fish captured during their staging period. The female captured on April 20, 2011 was likely going to spawn in the fall but possibly aborted the spawning run due to a long handling time. That same female returned to the James River 6 May 2013 and staged below rkm 67 from May to November except for two quick spawning movements, one to rkm 120 on 1 September and the other to rkm 132 on 24 September (Fig 3). Like many males (Fig 2), when the female returned to the James River in May of 2013 she moved up to rkm 67 before dropping back downstream to stage for the summer (Fig 3). Again, Coleman [33] described the AS fishing base operated around rkm 50. Looking at historical fishing locations and timing, this female could have been caught in April or May and would have been considered a spring spawn fish. To our knowledge monthly data are not available for historically fisheries in the James River. However, all colonial sources describe a large amount of AS during in the summer months which were likely fall-spawn fish and may have been the predominant spawning group. Further analysis of historical and recent data from other parts of the fish’s range suggest that the dual spawning is likely not limited to the James River.

**Fig 4:**
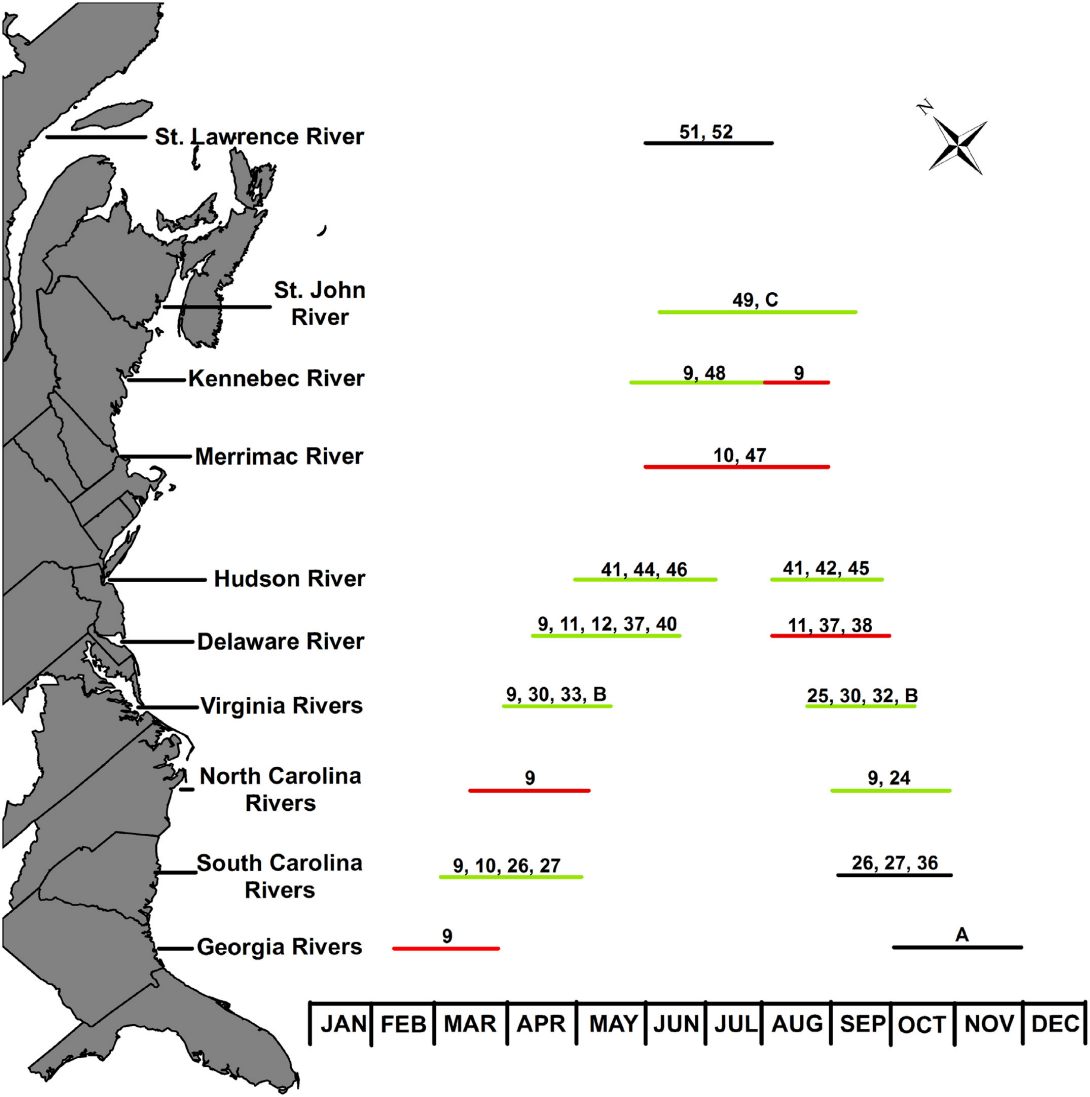
Map with bars showing spawning months of certain areas along the Atlantic sturgeon’s range. Red lines are spawning periods just described in historical records, black lines are relatively recent data, and green lines were described in both recent and historical records. Numbers refer to the references used to estimate spawning periods, letters are as follows: A, Peterson (Unpublished Data); B, This Paper; C, Cornel Ceapa (Personal Communication).

Georgia Rivers: Goode [10] described the AS fishery starting in the Satilla River in late February, and both adult males and females were harvested. Recent findings provide strong circumstantial evidence of AS spawning in the Altamaha River during October and November (Doug Peterson, University of Georgia, personal communication). The combination of historical and new data show there are probably two separate spawning races in the Georgia area, a February/March race and an October/November race (Fig 4). To our knowledge there are no recent data published supporting spring spawning still occurs Georgia Rivers.

South Carolina Rivers: Goode [10] described AS spawning runs in early March in the Edisto River. Goode [9] wrote that the spring fishery stopped around May in Winyah Bay. Leland [35] did an overview of the AS fishery in South Carolina and stated that commercial watermen reported spawning occurred after 15 May and before 1 August. In the Cooper River, the fishery peaked from 15 April-15 June, but breaching was observed from May to August. In the Santee River, AS were observed jumping during the last two weeks of May and in early August. Smith et al. [26] described AS commercial fisheries operated in the spring and fall. Using collections of adult fish and telemetry data, Collins et al. [27] described both spring and fall AS spawning in the Combahee and Edisto Rivers. Both ripe male and female sturgeon were taken in early March, and spent males were taken in late March with spent females taken as late as May. A different group of running males entered the river in late August into September and October. A developing female telemetered in early June was tracked on a run up into the spawning reaches in September. They also caught recently spent females in September and October. McCord et al. [36] showed a strong bimodal pattern of age-1 AS collected in the Edisto River. The bimodal age-1 pattern likely signifies spawning success during both spawning periods. Spring spawning fish of both sexes were larger on average than fall spawning fish [26, 27], similar to the pattern we observed in male fish caught in the James River. In South Carolina rivers the two spawning periods seem to be around April and October (Fig 4).

North Carolina Rivers: Goode [10] reported the Cape Fear River AS fishery was pursued during two periods, one from 10 March to the end of April and from 10 September to 1 November. Fish caught during the two periods were known as "New York Market" ripe. He also noted that spent sturgeon were taken during both the spring and the fall fisheries. The average catch per boat (n = 20) was about 50 fish during the summer months and 200 fish during the fall [10] suggesting an increase in AS numbers during the fall months. There are no descriptions of spring catch numbers. In 2013 fertilized AS eggs were collected during September in the Roanoke River [24]. Data suggest a March to April spring run and September to October fall run in North Carolina (Fig 4). Similarly to Georgia Rivers we are not aware of any published material suggesting spawning still occurs during the spring in North Carolina rivers.

Delaware River: Meehan [37] stated that early fisheries around 1840 targeted AS in July and August close to Philadelphia, and that the best way to get them was spearing around the fall line in August. Fall run AS in the James River move up to the fall line during August and September. Borodin [38] suggested that two spawning runs occur in the Delaware River with the spring run being bigger. Goode [10] noted that AS fishers targeted an area near Bower's Beach (Delaware Bay area) from April to May and later moved upriver. Ryder [11] noted that the AS fishery took place around Delaware City (near the salt wedge) as late as October. Borodin [38] noted catching post-spawn females in May. Ryder [1890] said sometimes fish were taken in the spring fishery with very immature roe, worthless for caviar (similar to our 20 April 2011 collection in the James River). Cobb [12] wrote that fish did not reach Chester, PA until 30 June which is when the fisheries were closed. The closure presumably was to protect fish spawning after that date. By 1925 the season closure was from 15 June to 15 August and was likely in place to protect females that had ripe eggs that were too soft to be processed in caviar [38]. Meehan [39] described catching females in July during the season closure that still had “hard roe”, the roe targeted for caviar but which was not yet mature nor suitable for fertilization. More information is needed detailing the timing of the ripening of hard roe. The presence of hard roe females in July suggests reproduction occurred much later than May. Breece et al. [40] using telemetry data described adults inhabiting the Delaware River from late April to late July. The situations may be similar in the James River and Delaware River in that historical fisheries likely targeted spring-spawn and staging fall-spawn fish throughout the summer months. If dual spawning occurs in the Delaware River we estimate the two spawning periods in the Delaware to be from April/June and August/September (Fig 4). To our knowledge there are no recent published data suggesting fall spawning occurs in the Delaware.

Hudson River, NY: In 1866 Lossing [41] noted that the Hudson River AS fishery focused around rkm 120 in Poughkeepsie from April to August. A newspaper article published in August 1878 described commercial fishers’ recent capture of gravid AS at rkm 132 [42]. In 1901 Townsend [43] described a new AS fishery targeting adult AS in the ocean off Suffolk County. Evermann [44] reported the new fishery described by Townsend [39] drifted 30.5–35.6 cm stretch mesh nets off Suffolk County during two seasons, May-June and September. The Suffolk County fishery was in the ocean around the mouth of the Hudson River so there is a chance the fish were coastal migratory fish. Evermann [44] noted the river fishery collapsed when the ocean fishery started.

In the 1970s post-spawn AS females were captured in May just above the salt wedge around rkm 64 and as the year progressed AS moved upstream, with the majority of spawning activity occurring between rkm 56 to rkm 132 during May to August [45]. Van Eenenaam et al. [46] reported capturing post-spawn and ovulatory females during June. Van Eenenaam et al. [46] also found two groups of adult female AS in the river during May/June, one group caught around rkm 55 which had immature eggs while the rest of the females were captured around rkm 135 and were either spawning or about ready to spawn. The relatively early egg stage females might have been staging in preparation for spawning during the late summer/early fall as they do in the James River. Considering that AS fisheries operated in freshwater from May through August [41, 43, 45], combined with the capture of non-ripe females in the lower river while another group was spawning up river [46], we suggest there are two spawning groups in the Hudson River. In the Hudson River we suggest the two spawning periods are from May/June and the other in August (Fig 4).

Merrimac River, MA: No information about actual spawning in the Merrimac River is available but the AS fishery has been reported to be in “summer” [9], and in August [47] (Fig 4).

Kennebec River, ME: Goode [10] reported a huge collapse in the Kennebec AS population after Edwards Dam was built at Augusta in 1837. AS came up the river in mid-summer as late as August and fishers “aimed to catch sturgeon early in the season, while roe was still black and hard, and to keep fish alive until the proper time arrived for opening them" [10], suggesting AS entered the rivers with very immature roe. Edwards Dam was removed in 1999. During 2009 through 2011, Wipplehauser [48] surveyed the Kennebec River using nets up to 30.5 cm stretch mesh. No females were captured during the Wipplehauser study. Successful reproduction was verified in 2011 by larva captures, one caught on 11 July and two on 12 July. Data from Wipplehauser [48] from telemetered adult males and larvae collections suggest some spawning occurs during June and July. Based on data from Wipplehauser [48] and Goode [10] AS reproduce in the Kennebec River from June through at least August, as either one extended spawning period or two separated by a small temporal gap (Fig 4).

Saint John River, NB: Rogers [49] reported that AS commercial fisheries were operated from July through September and fish were full of roe as late as 1 September. The overseer of the river district said spawning occurred from July through September, most occurring in August and September [49]. Current commercial fishers targeting AS capture post-spawn females as early as the first week of June and catch pre-spawn females in August (personal communication, Cornel Ceapa, Acadian Sturgeon and Caviar Inc.) Like in the Kennebec River, more research is needed to determine whether there is one extended spawning period or two separate groups, with one group spawning in June and the other group staging and spawning in August and September (Fig 4). Dadswell [50] suggests that the AS staying late into September overwinter in the river and spawn the next spring similarly to several Eurasian sturgeon species [14, 15, 16].

St. Lawrence River, QC: Scott and Crossman [51] reported that AS spawning occurred in late May to early July and added that many left the river from September to November. Hatin et al. [52] using telemetry data suggested spawning occurred in June and July (Fig 4). Like the St. Lawrence and Kennebec Rivers, more information is needed to determine if there is one spawning group or two groups separated by a short temporal gap.

Currently the James River AS spring-spawning race appears to be much less abundant than the fall race. The low numbers caught in the spring may be due to gear size or fishing methods. Three of the four male fish caught in the spring were larger than every confirmed male caught in the fall and the remaining spring male was larger than 94% of fall males. More effort using larger mesh sizes is required to better understand the spring race. Smith et al. [26] and Collins [27] described the same trend with spring AS being larger than fall fish. Other areas show differences among races of sturgeon in a single river. The hiemal Stellate Sturgeon that overwinter before spawning the following spring in the Ural River, Russia/Ukraine are smaller than the vernal run fish [14]. The hiemal Sterlet Sturgeon in the Volga and Kura Rivers have higher fecundity, grow larger, mature later, and live longer than the vernal race [15]. The two races in the James River may have varying life histories with the spring group either growing faster or maturing later compared to fall run fish.

The length-at-age curve developed by Balazik et al. [53] was based on juveniles in the lower James River and adults collected above rkm 105 during the fall-spawning run. We do not have any age information from known spring-run AS. Assuming a similar growth rate between spring and fall fish, all of the spring males were born prior to the AS moratorium established by the Atlantic States Marine Fisheries Commission in 1998, and 98% of the fall males were born after the moratorium. If fall-run males mature earlier than spring males it could be that we are seeing the results of the 1998 moratorium in the fall group first. The difference in size may have to do with the extended staging period of fall fish. Adult AS do not feed during spawning migrations [6] and males have up to a 90% annual return rate [28]. In the James River the duration of spring AS spawning period is less than two months compared to as much as seven months for the fall spawning period. If male AS fast during spawning runs fall males have considerable less time to feed in marine habitats compared to spring AS. Growth and age of maturity may be similar among the two groups but after maturity spring males may grow faster because they spend less time fasting compared to fall males.

It is also possible that the fall population has always been larger than the spring population. The peak of the James River historical harvest was in late May to July which is currently after the spring spawning season [33, 34]. It is plausible that a large proportion of the historical fishery harvested staging fall fish and the fall group was larger than the spring group. Staging fall adults would be more susceptible to fisheries considering the amount of time they spent in the river compared to the quick run of the spring fish. The two groups appear to utilize different portions of the rivers with the fall group spawning further upstream (Fig 2) similar to Eurasian species; this may be due to movement of the saltwedge. We would like to note that there were extensive shad (*Alosa sapidissima* and *A*. *mediocris*) fisheries in the James River during the 18^th^ and early 19^th^ centuries that often encountered AS. Because there was no market for AS at the time, AS were killed because they were very destructive to fishing gear [9, 10]. The timing of the shad run overlapped with spring-spawn AS so spring AS were being impacted by non-directed fisheries decades before the fall group. Due to bycatch in the shad fishery the spring group may have been somewhat depleted prior to the beginning of the directed AS fishery during the late 19^th^ century. Bycatch of AS in shad fisheries was common throughout the entire coast [9, 10].

Data suggest there are two races that repeatedly spawn during two different times and places in the James River and possibly the groups have become genetically distinct from each other. Over the past five years genetics from 139 fall run fish have been analyzed and 95% of those classify as James River AS based on the genetic population structure established by Tim King of the U.S. Geological Survey [54]. Only four known spring fish have been classified. Two males and one female classified as Delaware River fish and one male classified as a Hudson River fish. To our knowledge the one telemetered spring AS from 2012, classified as Delaware origin, has never moved into the Delaware or any other river; this fish has returned to the James River every spring during the life of the telemetry tag (Fig 2). There is a chance that all the spring fish caught and genetically analyzed were strays from other populations. However, we suggest that because of the relatively low number of spring run fish currently in the James River, their genetic signature may not have been represented when populations were determined. Because of the small sample size, it is premature to generalize about the genetic composition of the James River spring run AS. If there are two distinct groups in the James River and other rivers, population estimation based on genetic assignment may need to be modified.

The two cohorts spawning in the James River have different habitat utilization patterns within the river both temporally and spatially. Definition of these patterns is required to develop informed sampling and tagging protocols to better estimate population size. Furthermore, knowledge of extended spawning needs to be included in conservation and management measures that protect the species, *i*.*e*., dredging moratoria in the spring alone cannot be effective when most of the population is in the spawning reaches in the late summer and fall.

## Supporting Information

S1 Dataset This dataset shows the movements of selected fish throughout the study. The timeframe is the Julian Day broken up into four periods. The spring male was tagged in 2012 and returned in 2013 and 2014. The fall male was tagged in 2011 and returned each year after being tagged. The one female shown is a fall female that exhibits a long staging period in the lower part of the river prior to spawning.
(CSV)
